# Thalamic Injury and Cognition in Multiple Sclerosis

**DOI:** 10.3389/fneur.2020.623914

**Published:** 2021-02-05

**Authors:** Moein Amin, Daniel Ontaneda

**Affiliations:** ^1^Neurological Institute, Cleveland Clinic, Cleveland, OH, United States; ^2^Mellen Center for Multiple Sclerosis Treatment and Research, Neurological Institute, Cleveland Clinic, Cleveland, OH, United States

**Keywords:** cognitive impairment, multiple sclerosis (MS), MRI, neurodegeneration, thalamus

## Abstract

Multiple sclerosis (MS) produces demyelination and degeneration in both gray and white matter. Both cortical and deep gray matter injury is observed during the course of MS. Among deep gray matter structures, the thalamus has received special attention, as it undergoes volume loss in different MS subtypes and is involved in the earliest form of the disease, radiologically isolated syndrome. The thalamus plays an important role as an information relay center, and involvement of the thalamus in MS has been associated with a variety of clinical manifestations in MS, including fatigue, movement disorders, pain, and cognitive impairment (CI). Similar to thalamic volume loss, CI is seen from the earliest stages of MS and is potentially one of the most debilitating manifestations of the disease. The thalamus, particularly the dorsomedial nucleus as part of the basolateral limbic circuit and anterior thalamic nuclei through connections with the prefrontal cortex, has been shown to be involved in CI. Specifically, several cognitive performance measures such as processing speed and memory correlate with thalamic volume. Thalamic atrophy is one of the most important predictors of CI in MS, and both thalamic volume, diffusion tensor imaging measures, and functional activation correlate with the degree of CI in MS. Although the exact mechanism of thalamic atrophy is not well-understood, it is hypothesized to be secondary to degeneration following white matter injury resulting in secondary neurodegeneration and neuronal loss. The thalamus may represent an ideal biomarker for studies aiming to test neuroprotective or restorative therapies aimed at cognition.

## Introduction

Multiple sclerosis (MS) is one of the most common causes of neurological disability in young people, and cognitive impairment (CI) can be seen in 40–60% of patients (1, 2). CI can be seen in all causes of the disease, and MS commonly affects patients during peak years of productivity; thus, CI can lead to a significant burden for patients and society (1, 2). CI occurs despite a few patients with MS progressing to full-blown dementia (3). CI in patients with MS has been associated with reduced rates of employment, affecting the quality of life, independence, social participation, income, and access to health care (4, 5).

Although all cognitive domains may be affected in MS, the most commonly affected domains include learning and memory, speed of information processing, attention, and executive functioning, language, and social cognition (6–8). In patients with MS, predicting the severity of CI can be difficult. Some of the predictors for severity of CI in MS include greater age, disability score, cognitive reserve, sex, and possibly genetic markers (9, 10).

Lesion volume of demyelinated lesions in MS usually demonstrates a limited correlation with CI and cannot be used to accurately predict CI severity (11). Several other mechanisms regarding CI in MS include impairment of gray matter networks, atrophy of cortical and deep gray matter structures, including the thalamus (12, 13).

The thalamus has been implicated in CI in MS and several other neurological disorders. Reduced thalamic volume has been associated with CI in patients with epilepsy (14), dementia (15), stroke (16), and traumatic brain injury (17). In this article, we will review the role of the thalamus in cognition in MS.

## Thalamus and Cognition

Anatomically, the thalamus is composed of several different nuclei groups, including a lateral nuclear group, a medial nuclear group, anterior nuclear group, midline thalamic nuclei, reticular nucleus, and intralaminar nuclei (18). Functionally, the thalamus is classically divided into three groups, including the principal (relay) nuclei, association nuclei, and midline and intralaminar nuclei (19). Due to its integral function as a relay and integration center and taking part in several thalamocortical circuits, the role of the thalamus in cognition is well-recognized not only as a passive triage center but also contributing cognitive processes, including attention, speed of information processing, and memory (20).

The pulvinar nuclei are part of the lateral nuclei group and account for ~25% of the thalamic mass (21). Pulvinar nuclei have been shown to play a role in selective attention, as evident by increased glucose uptake during positron emission tomographic imaging (22). The anterior thalamic nucleus, through its connections to the hippocampus *via* the fornix and mammillothalamic tract, is associated with encoding content and contextual information and recollective processes (23, 24). The medial dorsal thalamic nucleus, with its connections to the prefrontal cortex and the limbic system through the basolateral limbic system, is related to executive aspects of memory, including strategic memory retrieval of information to be remembered and familiarity processes (23, 24). The intralaminar/midline thalamic nuclei, through their connections to the parietal lobe, play a role in attention, arousal, awareness, and activation of cortical regions necessary for the processing of information to be stored (23, 24). Disruption of thalamocortical white matter tracts has been shown to inversely correlate with cognitive domains such as verbal memory (25).

## Thalamus and Multiple Sclerosis

MS is a progressive inflammatory and neurodegenerative disease of the human CNS that leads to demyelination and neuronal/axonal loss, and both cortical and subcortical demyelination are observed during the course of MS, including gray matter structures such as the thalamus, hippocampus, caudate, putamen, globus pallidus, and other structures of the basal ganglia (26, 27).

### Imaging of the Thalamus in Multiple Sclerosis

T1- and T2-weighted images are less sensitive to detect lesions in gray matter regions due to inherent structural differences between gray matter and white matter and different inflammatory responses in those compartments (28–31). Thalamic lesions are usually more visible than cortical lesions, likely due to a higher density of myelin in the thalamus (32).

Thalamic lesions occur in two main types, subependymal or perivascular, and are present in 42–97% of patients (33–35). Imaging investigation in the thalamus have focused mainly on volumetric assessment (e.g., T1- or T2-weighted images, ultra-high field) and overall measures of thalamic integrity [e.g., susceptibility-weighted imaging (SWI), magnetization transfer ratio (MTR), magnetic resonance spectroscopy, diffusion tensor imaging (DTI)]. Common imaging modalities to detect thalamic pathology in MS are summarized in Table 1.

**Table 1:** Different MRI modalities and their use in the evaluation of thalamus and cognitive domains.

**Cognitive domain**	**Thalamic volume**	**DTI**	**SWI**	**MRS**	**UHF**
Verbal memory	Negative correlation with CVLT2 (36, 37)	Negative MD correlation with CVLT2 (36) Negative FA correlation (38)			
Visuospatial memory	Negative correlation with BVMTR (36, 37)	Negative MD correlation with BVMTR (36)	Negative pulvinar MP-LPV correlation with BVMTR (37)	Positive glutamate concentration correlation with PAL (39)	Negative thalamic volume and myelin density correlation with BVMTR (40)
Executive function (including attention and psychomotor speed)	Negative correlation with SDMT and PASAT (36, 37) Negative correlation with DKEFS (36, 37)	Negative MD correlation with SDMT, DKEFS, and PASAT (36) Negative FA correlation (38)	Negative pulvinar MP-LPV correlation with SDMT and DKEFS (37)		Negative thalamic lesion volume correlation with SDMT (34) Negative thalamic volume and myelin density correlation with DKEFS (40)

*UHF, ultra-high field; SWI, susceptibility-weighted imaging; DTI, diffusion tensor imaging; MRS, magnetic resonance spectroscopy*.

Different imaging modalities may be associated with different domains of CI. For example, in one study, diffusivity changes were consistently associated with information processing speed [Symbol Digit Modalities Test (SDMT)] and visual memory (Brief Visuospatial Memory Test—Revised), and verbal memory (California Verbal Learning Test, second edition,) but magnetic susceptibility was related only to SDMT performance (41).

Given the complex structural units within the thalamus, there has been growing interest in the evaluation of thalamic subnuclei and subregions. Subregion analysis of the thalamus has demonstrated an association of CI with mean diffusivity of a dorsomedial nucleus, orbitofrontothalamic tract, and amygdalothalamic tracts (42). In a recent study evaluating the association of thalamic nuclei volumes and cognitive performance, the volumes of anterior, medial, lateral, posterior, and ventral thalamic nuclei correlated positively with SDMT, whereas Brief Visuospatial Memory Test—Revised correlated with volumes of anterior, lateral, and medial nuclei (43). In another study, SDMT was correlated with superior and anterior volumes of the thalamus (44). Given the broad connections of the thalamus, the subregional analysis of different thalamic connections has also been evaluated. Reduced information processing speed was associated with atrophy of the bilateral frontal connected subregions, whereas SDMT negatively correlated with atrophy of frontal, motor, and connected temporal subregions (45). Functional MRI studies in MS have shown varying results. In one study, MS patients with cognitive impairment had increased resting-state functional connectivity between frontal, motor, occipital, and temporal subregions and hippocampus, parahippocampal gyrus, and superior temporal cortex (46). In another study, SDMT, paced auditory serial addition test, and California Verbal Learning Test all correlated with resting-state functional connectivity of several thalamic connections in healthy controls, but this association was not observed in MS patients (47).

### Thalamic Pathology

Clinically, thalamic involvement in MS manifests with a spectrum of diverse abnormalities, which range from fatigue and movement disorders to pain syndromes and cognitive decline. Given the broad range of the function of the thalamus, as described earlier, it is not surprising that thalamic involvement will have a wide variety of clinical manifestations in MS. Although the majority of evidence points to the thalamus as a site of secondary degeneration from distant white matter lesions, focal pathology within the thalamus may also, to some extent, affect function. Thalamic lesions have been found in around 71% of MS patients when imaged at 7 T (34, 35). The two main types of thalamic demyelinating lesions in MS include ovoid perivascular (perhaps due to extravasation of peripheral immune system cells from vasculature) and thin subependymal (perhaps due to diffusion of soluble toxin and chemokine infiltration from the CSF) lesions (33). In addition, histologically, dystrophic neurons, particularly in subependymal lesions (potentially excitotoxic injury), are observed, and the thalamic lesions contain a mixture of chronic active and inactive inflammation with or without demyelination (33).

Thalamic volume is inversely correlated with a physical disability and cognitive impairment in MS (33). There are likely several mechanisms for thalamic volume loss in MS. Progressive atrophy of the thalamus has been shown in all MS disease types (48), and loss of volume in the thalamus is one of the earliest and most prominent signs of deep gray matter pathology in patients with MS as seen in patients presenting with the clinically isolated syndrome (CIS) (49, 50) and radiologically isolated syndrome (51, 52). In another study, thalamic volume showed a weak relationship to physical disability score but was strongly correlated with cognitive performance in patients with MS (53). In a study evaluating the neuronal injury in the MS thalamus, authors found that both the thalamic volume and the concentrations of N-acetyl aspartate were decreased when compared with healthy controls (54). Thalamic neuronal density loss in MS (particularly smaller neurons) has been shown to occur at a faster rate compared with thalamic volume loss (30, 33). Further, the thalamic volume does not correlate with thalamic lesion volume, potentially implying that in addition to local inflammation, the volume loss could result from secondary neurodegeneration associated with the projecting tracts (30, 33). It should be noted that these studies were done at 3 T, and this association needs to be further studied using ultra-high field, where lesional pathology is more sensitively visualized. Retrograde degeneration can follow from focal white matter lesions in MS. The result of degeneration along white matter tracts could cause a neuronal loss in both cortical and deep gray matter structures, although this may not be the primary driving process in thalamic atrophy (55, 56). The impact of primary thalamic demyelination can potentially be better evaluated using MTR. MTR is a proposed marker of myelin content, and given the mixed nature of the thalamus as a white and gray matter, a structure may be more sensitive to this technique than other deep gray matter structures. MTR is decreased in the normal-appearing gray matter early in the disease course (mean duration of 1.9 years) and with mild clinical impairments (57).

DTI can detect changes compared with healthy controls in the normal-appearing thalamus, and the degree of thalamic changes correlate with functional impairment (31). White matter fractional anisotropy in the thalamus has been strongly associated with cognitive performance in MS patients (38). A DTI study examining thalamic connectivity using a stepwise regression analysis showed that thalamocortical lesion volume and the mean diffusivity in tracts connecting lesion and thalami were significantly correlated with thalamic volumes, which is a finding not observed in regions outside the thalamocortical white matter (50). In patients with primary progressive MS, thalamic volumetry and DTI measures have been shown to correlate with the extent of T2 hyperintense lesions as well as with the severity of microscopic damage to the normal-appearing white matter and normal-appearing gray matter, suggesting that both local inflammatory demyelination, as well as changes secondary to axonal transection of fibers passing through areas of diseased brain white matter, can account for thalamic abnormalities atrophy (58). DTI measures can also be used to improve the segmentation of the thalamus, which can allow a better evaluation of thalamic subnuclei in future studies (59). The role of iron in thalamic pathology is complex, as there have been mixed reports of iron content and concentration in MS, potentially due to different imaging techniques. In patients with MS, there is higher iron content compared with healthy controls (60), and increased thalamic susceptibility (proposed as iron content) using 7-T MRI has been seen in MS patients compared with healthy controls and associated with higher disability scores (61). This deposition may occur secondary to myelin and oligodendrocyte debris, as well as iron stores in macrophages, or it may be the product of hemorrhages from damaged brain vessels (62). Iron accumulation, particularly in the pulvinar, appears to be increased early in the course of the disease and reduced later in the course of the disease (63). Deposition of iron, as estimated by SWI, in the pulvinar nucleus of the thalamus has been shown to occur in the absence of volume change and atrophy, suggesting that this pathology may precede structure-specific atrophy (64). Increased iron accumulation in the early course of the disease can be potentially related to blood–brain translocation of heme-iron and microglial iron accumulation from higher perfusion supplied to thalamus compared with white matter, whereas the decrease in iron later in the course of the disease could potentially be related to reduction of oligodendrocytes density in thalamus (63). An alternative hypothesis suggests that increased iron concentrations may be the result of volume loss with a relatively fixed iron content, given the false impression of increased concentration (65).

## Longitudinal Thalamic Changes

The majority of the information regarding the role of thalamic injury in the MS disease process has been demonstrated using thalamic volume. A longitudinal study evaluating thalamic volumes in patients with primary progressive MS showed volume loss of thalami at baseline in MS patients compared with healthy control and further loss of volume after 1 year (66). A significantly reduced thalamic volume at baseline has also been associated with the development of CI in MS patients at 2-years follow-up (55). There is also evidence that the thalamus loses volumes at a different rate through the disease span. Compared with CIS, patients with relapsing and remitting MS display a faster rate of thalamic atrophy (67). In addition, a lower burden of white matter lesions and higher lifetime cumulative exposure to disease-modifying therapy have been shown to correlate with a slower rate of thalamic atrophy (67). Interestingly, this same group found that the thalamus had the most volume change from age 30–60 years but, after 60 years, showed a greater contribution from normal aging (68). This was not true in the caudate and putamen, further suggesting lesion accumulation drives thalamic volume loss.

When MS patients were followed for 15 months, thalamic DTI changes were found to be independent predictors of disability score deterioration. After 15 months, there was an increase in thalamic mean diffusivity and a decrease in thalamic fractional anisotropy (58). At 5-years follow-up, a reduction in fractional anisotropy of the anterior thalamic radiation can be used to predict CI in MS patients (69).

## Discussion

Thalamic involvement is an important feature of MS and can be seen early in CIS and radiologically isolated syndrome. Thalamus is a central relay structure with multiple connections and underpins a broad range of functions. As such, the involvement of the thalamus in MS can be associated with several neurological deficits, including CI. This involvement can be due to a combination of white matter and gray matter pathology as a result of direct inflammatory and cytotoxic damage as well as indirect neurodegeneration secondary to damage to its widespread afferent and efferent tracts. The histopathological hallmarks include neuronal loss, active and chronic inflammation with or without demyelination, and accumulation of iron deposits. Traditional MRI modalities may not be sensitive to detect thalamic changes, but several novel MRI modalities, including SWI, functional MRI, DTI, and magnetic resonance spectroscopy, have been used to evaluate thalamic pathology and their correlation with CI. With our improved understanding of the complex structure and connections within the thalamus, there is a need for better evaluation of these anatomical and connectivity subregions. Although there have been some recent advances in this area, there are limitations in spatial resolution, which could improve with advances in imaging techniques. There is also a paucity of research in histopathological changes in different thalamic subregions, which could be crucial in advancing our understanding of the pathophysiology of thalamic pathology. Furthermore, the thalamic pathology is an ongoing process throughout the course of the disease, although its rate can be affected by the activity of the disease. Many of the studies evaluating CI in MS are cross-sectional, and there are only limited reports studying the longitudinal changes in the thalamus and its subnuclei. There is a role for further research in understanding thalamic pathology, its progression, prevention, and targets for potential therapies for neurological restoration to prevent, delay, and reverse the impact of CI in MS patients.

## Author Contributions

MA and DO contributed to the design, literature review, analysis, and writing of the manuscript. All authors contributed to the article and approved the submitted version.

## Conflict of Interest

DO reports the following for support outside the current work: Research support from the National Institutes of Health, National Multiple Sclerosis Society, Patient Centered Outcomes Research Institute, Race to Erase MS Foundation, Genentech, Genzyme, and Novartis. Consulting fees from Biogen Idec, Genentech/Roche, Genzyme, Novartis, and Merck. The remaining author declares that the research was conducted in the absence of any commercial or financial relationships that could be construed as a potential conflict of interest.
